# Entomopathogenic nematodes from Mexico that can overcome the resistance mechanisms of the western corn rootworm

**DOI:** 10.1038/s41598-020-64945-x

**Published:** 2020-05-19

**Authors:** Pamela Bruno, Ricardo A. R. Machado, Gaétan Glauser, Angela Köhler, Raquel Campos-Herrera, Julio Bernal, Stefan Toepfer, Matthias Erb, Christelle A. M. Robert, Carla C. M. Arce, Ted C. J. Turlings

**Affiliations:** 1 Laboratory of Fundamental and Applied Research in Chemical Ecology, University of Neuchâtel, Emile-Argand 11, 2000 Neuchâtel, Switzerland; 2 Institute of Plant Sciences, University of Bern, Bern, Switzerland; 3 Neuchâtel Platform of Analytical Chemistry, University of Neuchâtel, Neuchâtel, Switzerland; 4 Present Address: Friedrich Schiller University Jena, Jena, Germany; 5 Instituto de Ciencias de la Vid y del Vino (Universidad de La Rioja, CSIC, Gobierno de La Rioja), Logroño, La Rioja Spain; 6 Department of Entomology, Texas A&M University, Texas, USA; 7 CABI, c/o Plant Protection and Soil Conservation Directorate, Hódmezővásárhely, Hungary

**Keywords:** Chemical ecology, Agroecology

## Abstract

Natural enemies of herbivores are expected to adapt to the defence strategies of their preys or hosts. Such adaptations may also include their capacity to cope with plant metabolites that herbivores sequester as a defence. In this study, we evaluated the ability of Mexican entomopathogenic nematodes (EPN) to resist benzoxazinoids that are sequestered from maize roots by the western corn rootworm (WCR, *Diabrotica virgifera virgifera;* Coleoptera: Chrysomelidae), an important maize pest in America and Europe. From maize fields throughout Mexico, we retrieved 40 EPN isolates belonging to five different species, with a majority identified as *Heterorhabditis bacteriophora*. In the laboratory, all nematodes readily infected non-sequestering larvae of the banded cucumber beetle (*D. balteata*), while infectivity varied strongly for WCR larvae. While some *H. bacteriophora* isolates seemed negatively affected by benzoxazinoids, most showed to be resistant. Thus, EPN from Mexican maize fields can cope with these plant defence metabolites, but the results also indicate that WCR larvae possess other mechanisms that help to resist EPN. This work contributes to a better understanding of the capacity of herbivore natural enemies to resist plant defence metabolites. Furthermore, it identifies several benzoxazinoid-resistant EPN isolates that may be used to control this important maize pest.

## Introduction

Biological control is a key element of integrated pest management. For the control of root feeding insect pests, some of the most effective natural enemies are entomopathogenic nematodes (EPN), which are generalist parasites of insects that inhabit the soils of most regions of the world^1–4^. They enter their hosts and regurgitate symbiotic bacteria that kill the insects by septicaemia and toxaemia^1,4^. The main EPN species used for biological control belong to the genus *Heterorhabditis* and *Steinernema*^2^, respectively associated to *Photorhabdus* and *Xenorhabdus* bacteria^5–7^. Both the EPN and their bacterial partners need to overcome the host defences for successful infection and propagation^8–13^. As a result of an evolutionary arms race with their natural enemies, insects have evolved a variety of defensive strategies to resist the attack of these enemies, one of which is to sequester secondary metabolites from the plants on which they feed^14–18^. The fact that the sequestration of plant-produced toxins by root-feeding insects can have a negative impact on the performance of EPN and their symbiotic bacteria has only recently become evident^19–21^.

The western corn rootworm (WCR, *Diabrotica virgifera virgifera* LeConte, Coleoptera: Chrysomelidae) is one of the pests that could be subject of biological control through EPN^22^. WCR is one of the main pests of maize in the United States Corn Belt^23,24^ and has been accidently introduced and spread throughout Europe since the 1980s^25^. The control potential of EPN is limited by the fact that WCR larvae are able to sequester benzoxazinoids^21^, the most abundant defence metabolites found in young maize tissue^21,26^. Contrary to generalist herbivores, which are negatively affected by benzoxazinoids^27^, WCR larvae themselves are immune and even attracted to benzoxazinoids^28,29^ and can convert and store them in their bodies^21^. Sequestration of benzoxazinoids has been shown to provide WCR larvae with resistance towards a commercial EPN strain and its symbiotic bacteria^21^.

Recent research found that EPN strains collected from US maize fields in regions where WCR has been present for at least 50 years were more effective in infecting WCR than EPN from other regions^30^. These EPN were less affected by benzoxazinoids sequestered by WCR, suggesting that EPN may be locally adapted to deal with the defence mechanisms of their insect hosts^30^. An artificial selection experiment found that benzoxazinoid resistance in EPN increases strongly when the nematodes were selected on WCR, and also increases significantly, but less strongly, when selected on the banded cucumber beetle (BCB, *D. balteata*), which does not sequester benzoxazinoids^30^. This latter result suggests that benzoxazinoid resistance in EPN may also increase in the absence of a benzoxazinoid sequestering host, possibly via exposure to residual benzoxazinoid levels in the herbivores or the rhizosphere.

WCR likely originated in Mexico^31^, where maize was domesticated *circa* 9000 years ago from teosinte (*Zea mays parviglumis*)^32^. Although WCR is present in Mexico, other root herbivores such as the larvae of the Mexican corn rootworm (*Diabrotica virgifera zeae*), the southern root cornworm (*D. undecimpunctata*), the banded cucumber beetle (*D. balteata*) and white grubs, such as *Phyllophaga* spp., are often abundant in maize fields^33–35^. No information is currently available on the capacity of Mexican maize root pests other than WCR to sequester benzoxazinoids. In this study, we explored the capacity of EPN strains that were isolated from Mexican corn fields to infect BCB and WCR and resist benzoxazinoids. We hypothesized that Mexican EPN should have an enhanced capacity to resist the benzoxazinoid-dependent defences of WCR due to the fact that they evolved in a benzoxazinoid-rich environment and may have shared an evolutionary history with benzoxazinoid containing root herbivores. To this end, we isolated and identified 40 EPN isolates from maize fields throughout Mexico. We then assessed their ability to infect WCR and BCB in the laboratory. In a second step, we tested the impact of benzoxazinoids on the resistance of WCR towards the different EPN isolates by feeding WCR larvae on benzoxazinoid-containing or benzoxazinoid-deficient maize seedlings. Taken together, these experiments allowed us to assess the capacity of different Mexican EPN to infect and kill two different rootworm species and to gain insights into the prevalence and relative importance of benzoxazinoid resistance in these EPN isolates.

## Results

### Mexican maize fields host several EPN species

**Figure 1:**
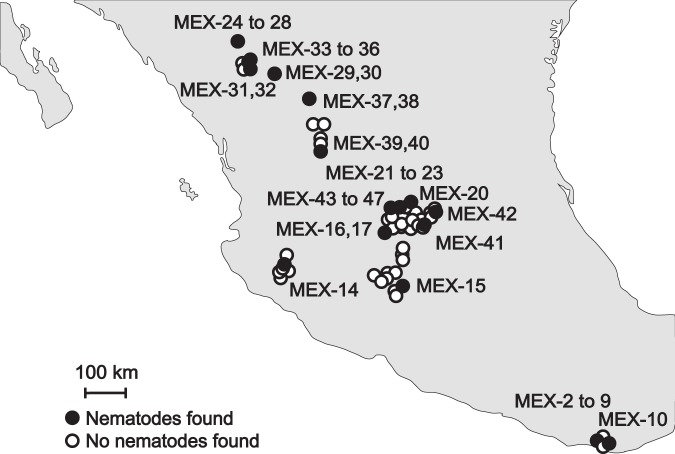
Location of soil samples and origin of entomopathogenic nematode isolates (EPN) obtained from 16 Mexican maize fields out of 114 maize fields sampled. One mixed soil sample was taken per maize field location and baited with *Galleria mellonella* larvae. Black filled dots indicate sites where EPN isolates were collected. Empty dots indicate soil samples from which no nematodes were obtained. Original map by FreeVectorMaps.com, modified with Adobe Illustrator CC 2018.

**Figure 2:**
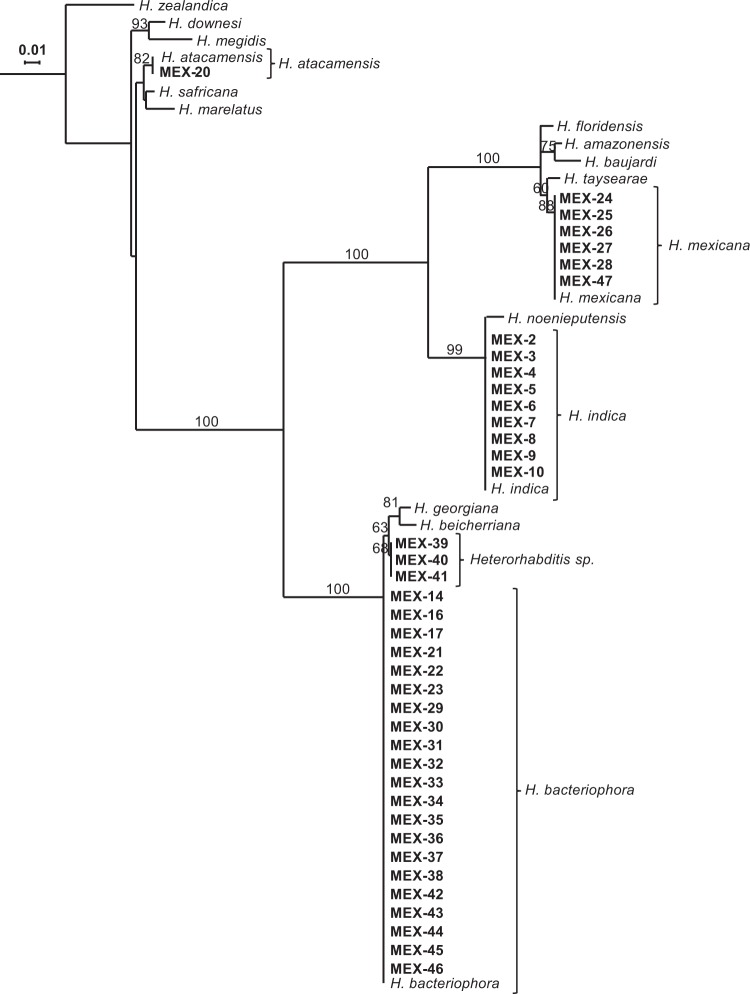
Maximum-likelihood phylogenetic tree of *Heterorhabditis* nematodes based on 18S ribosomal RNA gene sequences. The evolutionary distances were computed using the Hasegawa-Kishino-Yano model. The tree with the highest log likelihood (−3422.19) is shown. Numbers at nodes represent bootstrap values higher than 70% based on 100 replications. The rate variation among sites was modeled with a gamma distribution (5 categories (+G, parameter = 0.4803)). Bar represents 0.01 nucleotide substitutions per sequence position. Accession numbers of the gene sequences used for the reconstructions and those obtained in this study are available in Tables S1 and S2, respectively.

**Figure 3:**
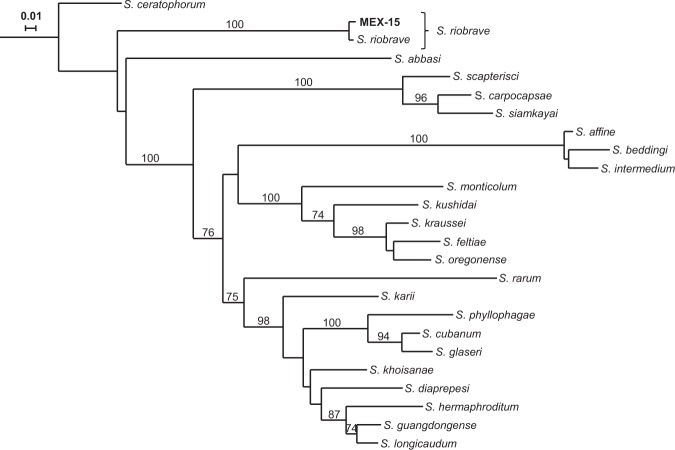
Maximum-likelihood phylogenetic tree of *Steinernema* nematodes based on 18S ribosomal RNA gene sequences. The evolutionary distances were computed using the General Time Reversible model. The tree with the highest log likelihood (−7942.12) is shown. Numbers at nodes represent bootstrap values higher than 70% based on 100 replications. The rate variation among sites was modeled with a gamma distribution (5 categories (+G, parameter = 0.4233)). Bar represents 0.01 nucleotide substitutions per sequence position. Accession numbers of the gene sequences used for the reconstructions and those obtained in this study are available in Tables S1 and S2, respectively.

In total, 40 EPN isolates were retrieved by baiting method from mixed soil samples of 16 out of 114 maize fields (14%) from the states of Oaxaca, Jalisco, Michoacán, Guanajuato, Zacatecas and Durango (Fig. 1, Table 1). Durango state had the highest percentage of EPN-hosting maize fields (44%, 4 out of 9). The other locations had 8 to 18% positive samples, except Querétaro locations, from which no EPN were collected. According to 18S, D2/D3 and concatenated 18S-D2/D3 rRNA gene sequences comparisons, the recovered EPN belong to the species *Heterorhabditis indica*, *H. atacamensis*, *H. mexicana*, *H. bacteriophora, Steinernema riobrave* and a potentially new species closely related to *H. bacteriophora* (Figs. 2,3, Supplementary Figs. S1–S8). Independently of the evolutionary inference method (Maximum Likelihood or Neighbour Joining) or the sequence used, similar taxonomic conclusions can be drawn (Figs. 2,3, Supplementary Figs. S1–S8). *Heterorhabditis bacteriophora* was the most dominant species (21 of 40 isolates, 52.5%) and its distribution was the broadest, being recovered in Durango, Zacatecas, Jalisco and Guanajuato states. Three isolates, MEX-39, MEX-40, MEX-41, form a clearly distinct cluster from the close *H. bacteriophora*, and might therefore be a new species (Fig. 2, Supplementary Figs. S1-S2 and S3-S4). Eight isolates of *H. indica* were recovered, all from Oaxaca state, a single *S. riobrave* isolate was obtained from Michoacán state, and a single *H. atacamensis* isolate and most of the *H. mexicana* isolates were recovered from Guanajuato state.

**Table 1:** Isolates obtained per entomopathogenic nematode-infected sample and origin of soils of Mexican maize fields. Each *Galleria mellonella* larva from the ‘*Galleria* baiting” soil extraction method was kept separately and the emerging EPN formed one isolate.

State	Sampled fields	EPN infected fields	Entomopathogenic nematodes			
			Latitude (°)	Longitude (°)	Altitude (masl)	Isolates
Oaxaca	19	2	15.886541	−97.116045	1	MEX-2, MEX-4, MEX-5, MEX-6,MEX-7, MEX-8, MEX-9
			15.920639	−97.12642	20	MEX-10
Jalisco	13	1	19.893941	−104.04043	903	MEX-14
Michoacán	13	1	19.627714	−101.41115	2277	MEX-15
Guanajuato	45	6	20.738882	−101.33939	1760	MEX-16, MEX-17
			20.963636	−100.83139	1869	MEX-20
			20.924801	−100.94449	1993	MEX-21, MEX-22, MEX-23
			20.470774	−100.59571	1926	MEX-41
			20.565299	−100.77389	1766	MEX-42
			20.914769	−101.21872	1913	MEX-43, MEX-44, MEX-45, MEX-46, MEX-47
Querétaro	4	0	—	—	—	—
Zacatecas	11	2	23.208545	−103.04567	2080	MEX-37, MEX-38
			22.161371	−102.88794	1743	MEX-39, MEX-40
Durango	9	4	24.006255	−104.61634	1789	MEX-24, MEX-25, MEX-26, MEX-27, MEX-28
			24.000478	−104.61628	1878	MEX-29, MEX-30
			23.954223	−104.62634	1885	MEX-31, MEX-32
			23.988059	−104.62605	1880	MEX-33, MEX-34, MEX-35, MEX-36
TOTAL	114	16

### Contrary to BCB, WCR larvae sequester benzoxazinoids from maize seedlings

**Figure 4:**
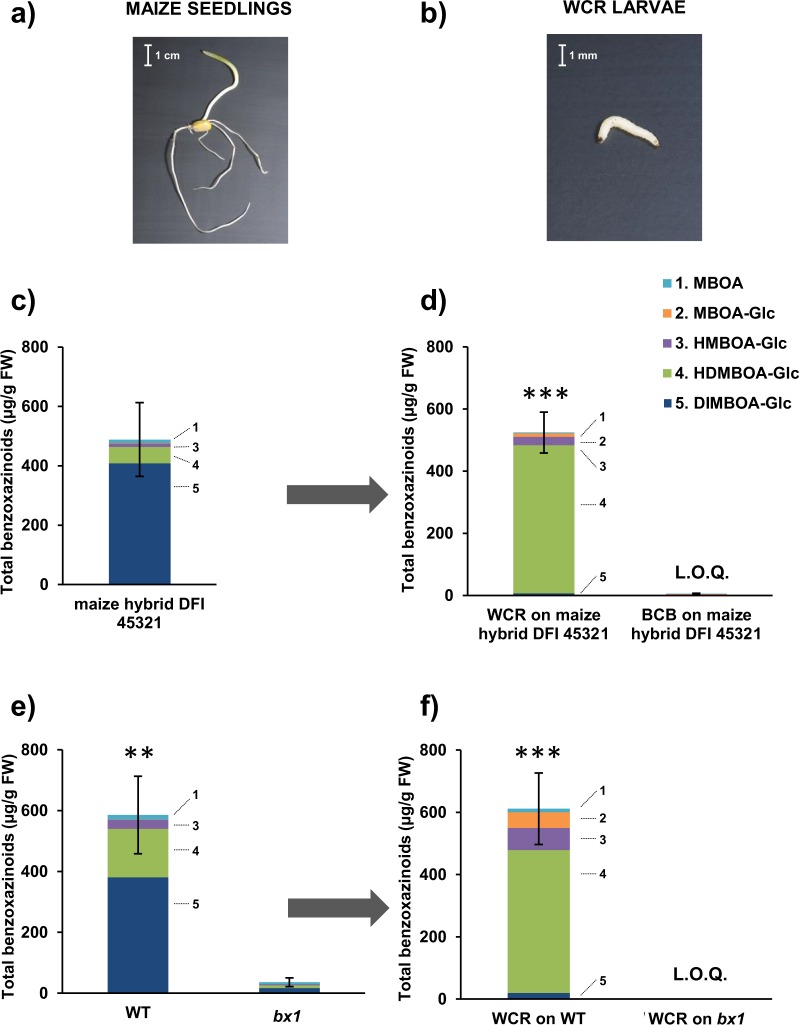
Contents of benzoxazinoids in maize seedlings and sequestration of benzoxazinoids by western corn rootworm larvae. (**a**) Maize seedlings used for the rearings of WCR and BCB. (**b**) Larvae of WCR used in the experiments. (**c**) Benzoxazinoids concentrations in maize seedlings of the hybrid DFI 45321 (n = 5). (**d**) Benzoxazinoids concentrations in dissected larvae (n = 5) of WCR and BCB fed on hybrid maize seedlings DFI 45321. (**e**) Benzoxazinoids concentrations in maize seedlings (n = 5) of the benzoxazinoid-containing wild type (WT) B73, and the benzoxazinoid-deficient *bx1* mutant maize. (**f**) Benzoxazinoids concentrations in dissected WCR larvae fed on the WT B73 (n = 4) or on the benzoxazinoid-deficient *bx1* mutant (n = 5) since hatching. Bars indicate average (± SE). Stars indicate significant differences between treatments: ***p < 0.001, **p < 0.01.

To test whether WCR larvae sequester benzoxazinoids under our experimental conditions, we quantified the benzoxazinoids present in seedlings of maize and in larvae fed on those seedlings (Fig. 4a,b). We first quantified the benzoxazinoids in the seedlings of DFI 45321 maize, which is the hybrid used as insect food in our *in-house* rearing, as well as in WCR larvae that fed on that maize. The total amount of benzoxazinoids found was 489 ± 125 µg/g fresh weight (FW) in DFI 45321 maize seedlings, of which 84% was DIMBOA-Glc and 11% was HDMBOA-Glc (Fig. 4c). WCR larvae fed on seedlings of this maize contained 525 ± 66 µg/g FW of benzoxazinoids, mainly composed of HDMBOA-Glc (91%) (Fig. 4d, Supplementary Table S3). MBOA-Glc was not present in plants, though it was present in larvae, which confirms previous findings^36,21^. BCB larvae fed on DFI 45321 maize, on the other hand, had <1% of the total amount of benzoxazinoids found in WCR larvae (Fig. 4d, Supplementary Table S3, F2,10 = 65.585, p < 0.001).

We then measured benzoxazinoids in maize seedlings of wild type B73 and in a corresponding benzoxazinoid-deficient *bx1* mutant line. We found 586 ± 127 µg/g FW of benzoxazinoids in wild type B73 seedlings, of which, DIMBOA-Glc represented 65% and HDMBOA-Glc 27% (Fig. 4e, Supplementary Table S4). In contrast, the maize seedlings of the *bx1* mutant had 95% less total benzoxazinoids content compared to wild type B73 maize (Fig. 4e, Supplementary Table S4, F2,10 = 19.922, p = 0.002). WCR larvae that were fed on wild type B73 maize seedlings had a total benzoxazinoid content of 612 ± 115 µg/g FW, 75% of which consisted of HDMBOA-Glc, whereas WCR larvae fed on *bx1* mutant seedlings had only 0.05% of the total benzoxazinoids found in WCR larvae fed on the wild type B73 maize (Fig. 4f, Supplementary Table S5, F2,9 = 45.328, p < 0.001). Together, these results confirm earlier findings that WCR, but not BCB, sequesters benzoxazinoids.

### Mexican EPN show high variability in infecting WCR but not BCB larvae

**Figure 5:**
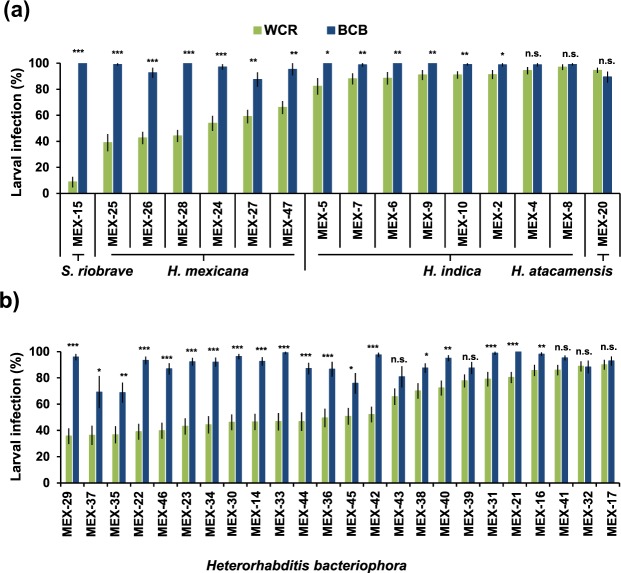
Infection rates of all Mexican EPN isolates on WCR larvae (green) and BCB (blue) seven days after EPN inoculation. (**a**) *Steinernema riobrave*, *Heterorhabditis mexicana*, *H. indica* and *H. atacamensis*, and (**b**) *Heterorhabditis bacteriophora* EPN isolates (n~20). Bars indicate average (±SE). Stars indicate significant differences between treatments for each EPN isolate: *p < 0.05, **p < 0.01, ***p < 0.001.

In order to screen the capacity of the collected EPN isolates to infect the benzoxazinoid-sequestering WCR larvae, we compared the infection capacity of each EPN isolate towards benzoxazinoid-sequestering WCR larvae and non-sequestering BCB larvae. Infection capacity across different EPN isolates appeared highly variable on WCR larvae, but was generally high and homogeneous on BCB larvae (Fig. 5, F_2, 2111_ = 543.57, p < 2.2E-16). *Steinernema riobrave* nematodes infected few WCR (9% ± 4) but all BCB larvae (100%) (Fig. 5a). In contrast, *H. indica* and *H. atacamensis* nematodes were highly infective on both, WCR (90 ± 4% and 95 ± 2%, respectively) and BCB larvae (99% ± 1% and 89 ± 4% respectively) (Fig. 5a). *Heterorhabditis mexicana* isolates were moderately infective on WCR larvae (51 ± 5%), but strongly infective on BCB larvae (95 ± 3%) (Fig. 5a). The most variable infection rates were found in *H. bacteriophora* isolates on WCR larvae, which varied from 35 ± 6% to 90 ± 4% (Fig. 5b). Thus, WCR larvae are more resistant to most Mexican nematode isolates than BCB larvae. Results of the statistical analyses are shown in Supplementary Table S6.

### Most *Heterorhabditis bacteriophora* isolates are adapted to the benzoxazinoid-dependent defences of WCR

**Figure 6:**
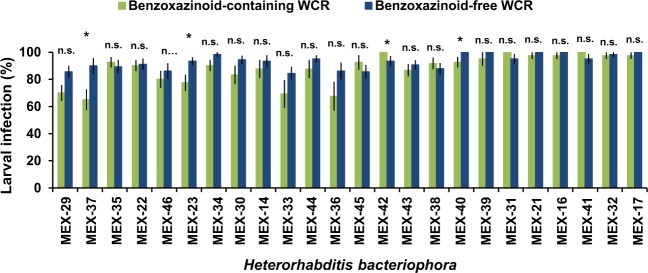
Infection rates of *Heterorhabditis bacteriophora* isolates on WCR larvae fed on benzoxazinoids-containing (green) or benzoxazinoid-free (blue) maize seedlings seven days after EPN inoculation. Bars indicate average (±SE). Stars indicate significant differences between treatments for each EPN isolate: *p < 0.05, **p < 0.01, ***p < 0.001.

The high variation in the infection capacity on WCR larvae across EPN isolates, compared to the overall-high infection on BCB, suggested that the nematode isolates differed in their capacities to resist benzoxazinoid-dependent WCR defence. In order to test this hypothesis, we evaluated the infection capacity of all *H. bacteriophora* isolates on benzoxazinoid-containing and benzoxazinoid-free WCR larvae. To produce benzoxazinoid-free WCR larvae, we fed them on benzoxazinoid-deficient *bx1* mutant maize seedlings. The infection capacity was uniform across all *H. bacteriophora* isolates against both benzoxazinoid-containing and benzoxazinoid-free WCR larvae, except the isolates MEX-37, MEX-23 and MEX-40, which showed a significantly lower infection rate on benzoxazinoid-containing larvae than their benzoxazinoid-free counterparts (Fig. 6). Surprisingly, the infection of benzoxazinoid-containing WCR larvae by the isolate MEX-42 was higher than of benzoxazinoid-free WCR larvae. Results of the statistical analyses are shown in Supplementary Table S7.

## Discussion

For the present study, 40 novel EPN isolates were obtained after surveying114 maize fields across seven Mexican states. Using phylogenetic reconstructions based on rRNA gene sequences, they were identified as *Heterorhabditis indica*, *H. atacamensis*, *H. mexicana*, *H. bacteriophora*, and *Steinernema riobrave*. Interestingly, the *H. bacteriophora*-classified MEX-39, MEX-40 and MEX-41 isolates form a slightly, but clearly distinct group from *H. bacteriophora*. Traditionally, isolates sharing more than 97% similarity in rDNA sequences are classified as same species, which applies to MEX-39, MEX-40 and MEX-41. However, it is increasingly recognized that the 97% threshold is rather conservative and recent studies suggest that the threshold for species delimitation can be greater than 99%^37–39^.Therefore, we should be open to the possibility that the isolates MEX-39, MEX-40 and MEX-41 represent a new species. Further evidence based on cross-breeding analyses should be obtained to test this possibility^36^. Similar results with Rwandan isolates close to or the same as *H. bacteriophora* were recently reported by Fallet *et al*.^40^

Plant chemical defences can be exploited by adapted herbivores^41^. A prime example is the sequestration of plant defence compounds by herbivorous insects, which help them to protect themselves against their natural enemies^15,17,21,42,43^. However, natural enemies may adapt to overcome the toxic effect of plant secondary metabolites that they frequently encounter in their environment. It has recently been postulated that, although EPN are often generalists^44^, their limited mobility confines populations to small geographic areas, and that this confinement exerts sufficient pressure to each population to adapt to local hosts and associated plant secondary metabolites^30^. Thus, EPN populations that have coexisted with benzoxazinoids for a long time are expected to have evolved some tolerance or resistance to these compounds. Indeed, when exposed to WCR fed either on benzoxazinoid-containing or benzoxazinoid-deficient maize seedlings (Fig. 6), almost all of our Mexican *H. bacteriophora* isolates were equally capable to kill both types of larvae. This is in agreement with the findings of Zhang *et al*.^30^ and implies that most of *H. bacteriophora* are adapted to benzoxazinoid-dependent defences of WCR. Interestingly however, we still observed substantial variability among the isolates in their capacity to infect WCR, suggesting the presence of other WCR defence mechanisms to which not all EPN are equally well adapted. This variability may also be due to the fact that some isolates are somewhat distinct from *H. bacteriophora*, and may even represent a new species.

As WCR is usually not very abundant in Mexican maize fields^35^, EPN populations may vary in their ability to overcome WCR defence mechanisms that are independent of benzoxazinoids. Evolved resistance to sequestered benzoxazinoids might only be one of the strategies that renders some but not all the EPN isolates highly infective on WCR. Our results imply that EPN and/or their symbiotic bacteria have also evolved other traits to overcome WCR resistance. They may, for instance, be able to suppress the humoral immune response of the host insect^45^. This ability often depends on the EPN-bacteria association and indeed has been reported to differ even among conspecific EPN strains^46,47^. Additionally to the effects of sequestered benzoxazinoids, symbiotic bacteria may also be affected by other factors in the host’s diet, to which they could evolve resistance^19,21^. It should be noted that WCR larvae in this study were fed with different maize genotypes in two experiments, and nutritional or chemical aspects of DFI 45321 and B73 maize may have affected the susceptibility of WCR larvae to EPN infection^20,48^. Possibly, WCR larvae acquired a resistance factor from DFI 45321 maize that is absent in B73 maize, which could help explain the overall high infection of WCR larvae in the second infectivity experiment. Our work indicates that WCR larvae possess effective defence strategies, much more than BCB larvae, that allow them to escape certain EPN which have overcome benzoxazinoid sequestration.

The potential of *H. bacteriophora* as a biological control agent of WCR has been widely recognized and studied, and commercial products based on this EPN are registered in a number of European countries. It is considered as one of the most effective EPN species in the biological control of WCR larvae, particularly in light of the recent phase-out of neonicotinoid-based seed coatings and highly toxic soil pesticides such as tefluthrin^49–51^. Nevertheless conventional EPN application methods have various limitations such as being knowledge-intense for the farmer^52^, or sometimes not cost-effective for low-value crops like maize. But novel, more effective EPN application methods are under development, such as application in alginate beads^53–55^. In addition, our results show that selecting the *H. bacteriophora* most adapted to benzoxazinoid-based defences of WCR or breeding for such traits may improve the biocontrol product efficacy. It may also be wise to include the so far little-considered *H. indica* or *H. atacamensis* in research and development on the biological control of WCR. In conclusion, readily mass-produced^56^, highly-infectious EPN that are well adapted to overcome WCR resistance could be the best candidates for further improving biocontrol strategies against this exceedingly important maize pest.

## Materials and methods

### Biological resources

Maize (*Zea mays mays* L.) of the inbred line B73 (wild type) and the near-isogenic line *bx1*^57^ (*bx1* mutant) were provided by the laboratory of Biotic Interactions (University of Bern, Switzerland). Seeds were germinated in humid vermiculite (Greendoor, Switzerland) and 5-day-old seedlings were used for the rearing of the larvae.

WCR eggs were originally provided by CABI (Hódmezővásárhely, Hungary) from the WCR non-diapause colony of USDA ARS (Brookings, SD, USA). BCB eggs were provided by Syngenta Crop Protection (Stein, Switzerland). WCR and BCB were reared on the maize hybrid DFI 45321 (DSP, Delley, Switzerland) as previously described^58^. Briefly, neonate larvae were placed on 5-day-old germinated maize seedlings in rearing boxes (BugDorm, USA) filled with potting soil (Einheitserdewerke Patzer Gebrüder Patzer GmbH & Co. KG, Germany), and the plant material was refreshed every three days. The adults were kept in cages (BugDorm, USA) and fed with 10-day-old maize plants. Neonates of WCR and BCB were fed on either the maize hybrid DFI 45321, the wild type B73 or the mutant line *bx1* until the larvae reached the third instar and then they were used for the experiments. All experiments were performed using third instar larvae.

### Survey of entomopathogenic nematodes

Entomopathogenic nematodes were surveyed in 114 maize fields in the Mexican states of Durango, Zacatecas, Guanajuato, Querétaro, Michoacán, Jalisco and Oaxaca, during July and August 2012 (Table 1). Four locations were randomly selected in each field to collect 500 g at 20 to 40 cm depth. The four samples were homogenized and pooled to obtain one 2 kg representative soil sample per field. EPN were recovered using the “*Galleria* baiting” method^59,60^. Briefly, two plastic pots of 250 ml were filled with soil from each soil sample and ten larvae of the greater wax moth *Galleria mellonella* L. (Lepidoptera: Pyralidae) were added. The pots were covered and kept at room temperature in the dark for four days, after which the mortality of the larvae was evaluated. Dead and living larvae were removed, and the soil was baited for a second time with 10 fresh larvae. The dead larvae were individually placed onto paper in nematode traps, consisting of islands of small Petri-dishes in a water filled larger Petri-dish^61^. Emerging infective juveniles from each larva were separately collected from the water. *In vivo* colonies of EPN were established under quarantine conditions at the University of Neuchâtel, Switzerland (nematode storage in darkness at 12 °C).

### Identification of entomopathogenic nematodes

Genomic DNA was extracted using the QIAamp DNA Mini Kit, following manufacturer’s instructions (Qiagen Ltd., Valencia, CA). For this, 20,000 freshly emerged infective juveniles were centrifuged for ten minutes at 4 °C and 10,000 G. The supernatant was removed, and the nematode were resuspended in 200 µl of milli-Q water in a 1.5 ml microcentrifuge tube. The nematodes were then ground for 15 seconds with a pestle motor, 180 µl of buffer ATL and 20 µl of proteinase K (600 mg/ml) were added, and the tubes were incubated at 56 °C for 2 hours. The extracted DNA was eluted in 50 µl of milli-Q water and stored at −20 °C until analysis. The quality of the DNA was evaluated with nanodrop and electrophoresis migration. PCR was performed to amplify the 18S rRNA gene using primers 18S (5′-TTGATTACGTCCCTGCCCTTT-3′) and 28S (5′-TTTCACTCGCCGTTACTAAGG-3′)^62^ and the D2/D3 region of the 28S rRNA gene using primer 536 (5′-CAGCTATCCTGAGGGAAAC-3′)^63^. PCR products were purified using the QIAquick gel purification Kit (Qiagen Ltd., Valencia, CA) and sequenced by Sanger sequencing (Microsynth AG, Balgach, Switzerland). The evolutionary histories were inferred from either the 18S, the D2/D3 or both sequences concatenated using the Neighbour-joining method^64^ and the Maximum-Likelihood method^65^. For Maximum-Likelihood method-based phylogenies, best-fit substitution model analysis were carried out prior to inferring evolutionary histories^66,67^. All trees are drawn to scale, with branch lengths measured in the number of substitutions per site. The percentage of trees in which the associated taxa clustered together is shown next to the branches. In all cases, initial tree(s) for the heuristic search were obtained automatically by applying Neighbor-Join and BioNJ algorithms to a matrix of pairwise distances estimated using the Maximum Composite Likelihood (MCL) approach, and then selecting the topology with superior log likelihood value. The Maximum-likelihood *Heterorhabditis* 18S-based evolutionary history was inferred based on the Hasegawa-Kishino-Yano model^68^. The tree with the highest log likelihood (−3422.19) is shown. A discrete Gamma distribution was used to model evolutionary rate differences among sites (5 categories (+G, parameter = 0.4803)). There were a total of 849 positions in the final dataset (Fig. 2). The Maximum-likelihood *Heterorhabditis* D2/D3-based evolutionary history was inferred based on the Kimura 2-parameter model^69^. The tree with the highest log likelihood (−1812.68) is shown. A discrete Gamma distribution was used to model evolutionary rate differences among sites (5 categories (+G, parameter = 0.0500)). There were a total of 845 positions in the final dataset (Supplementary Fig. S1). The Maximum-likelihood *Heterorhabditis* concatenated 18S-D2/D3-based evolutionary history was inferred based on the Kimura 2-parameter model^69^. The tree with the highest log likelihood (−6209.06) is shown. A discrete Gamma distribution was used to model evolutionary rate differences among sites (5 categories (+G, parameter = 0.2726)). There were a total of 1892 positions in the final dataset (Supplementary Fig. S2). The Maximum-likelihood *Steinernema* 18S-based evolutionary history was inferred based on the General Time Reversible model^66^. The tree with the highest log likelihood (−7942.12) is shown. A discrete Gamma distribution was used to model evolutionary rate differences among sites (5 categories (+G, parameter = 0.4233)). There were a total of 832 positions in the final dataset (Fig. 3). The Maximum-likelihood *Steinernema* D2/D3-based evolutionary history was inferred based on the Kimura 2-parameter model^69^. The tree with the highest log likelihood (−4491.90) is shown. A discrete Gamma distribution was used to model evolutionary rate differences among sites (5 categories (+G, parameter = 0.2931)). There were a total of 879 positions in the final dataset (Supplementary Fig. S3). The Maximum-likelihood *Steinernema* concatenated 18S and D2/D3-based evolutionary history was inferred based on the Kimura 2-parameter model^69^. The tree with the highest log likelihood (−16814.12) is shown. A discrete Gamma distribution was used to model evolutionary rate differences among sites (5 categories (+G, parameter = 0.3552)). There were a total of 2126 positions in the final dataset (Supplementary Fig. S4). For Neighbour-Joining based phylogenies, the evolutionary distances were computed using the Kimura 2-parameter model^69^, with gamma-distributed rate variation among sites. The trees were constructed with the bootstrap test (1000 replicates) showing the percentage of replicate trees in which the associated taxa clustered together^70^ (Supplementary Figs. S5–S8). Interactive Tree of Life v3.5.1^71,72^ was used to edit and represent the phylogenetic trees. All evolutionary analyses were conducted in MEGA7^67^. All the sequences obtained were deposited into the GenBank. Accession numbers of the gene sequences used for the reconstructions and those obtained in this study are available in Tables S1 and S2, respectively.

### Benzoxazinoids profiling in insects and plants

To assess benzoxazinoid sequestration by the insect larvae, we measured the content of benzoxazinoids in larval tissues of third instar WCR and BCB larvae fed on hybrid DFI 45321 maize seedlings, as well as WCR larvae fed either on the wild type B73 or on *bx1* mutant maize. As previously reported, the sequestration occurs when the chemical compounds are transferred from the gut to other body tissues, such as muscles and haemolymph^15,16,18^. Therefore, the benzoxazinoids were measured from larvae from which the gut was removed (n = 5). Eight to ten larvae were pooled per biological replicate. We also quantified the amount of benzoxazinoids in the maize seedlings of the genotypes that were used in the experiments (n = 5, five days old). Maize seedlings or larvae (guts removed) were flash frozen in liquid nitrogen and ground into a fine powder. To 100 mg of plant or 20 mg of larval tissue per sample, 1 ml or 400 µl of the extraction buffer (MeOH: H_2_O: formic acid (FA); 50: 50: 0.5%) were added, respectively. Extracts were vortexed for 1 min and centrifuged at 20,000 g for 20 min at 4 °C. Supernatants were used for further analyses. Plant samples of the hybrid DFI 45321 and the wild type B73 were diluted 50 times, and samples of the the maize mutant *bx1* were diluted 20 times. Larval extracts were directly used without dilution for the analyses. We measured the main benzoxazinoids that had previously been found in maize plants and in WCR larvae: 2-(2-hydroxy-4,7-dimethoxy-1,4-benzoxazin-3-one)-β-d-glucopyranose (HDMBOA-Glc), 2-(2,4-dihydroxy-7-methoxy-1,4-benzoxazin-3-one)-β-d-glucopyranose (DIMBOA-Glc), 2‐β‐d‐glucopyranosyloxy‐7‐methoxy‐1,4‐benzoxazin‐3‐one (HMBOA-Glc), 6-methoxy-2-benzoxazolinone N-glucoside (MBOA-Glc) and 6-methoxy-2-benzoxazolinone (MBOA)^21,28,73–75^. The quantification was performed by ultra-high-performance liquid chromatography tandem mass spectrometry (UHPLC-MS/MS). The equipment was composed of an Ultimate 3000 RSLC (Dionex, Thermo Fisher Scientific) interfaced to a 4000 QTRAP (AB Sciex) through an electrospray probe. Benzoxazinoids were separated on an Acquity UPLC BEH C18 column (50 × 2.1 mm, 1.7 μm, Waters) in gradient mode using H_2_O + formic acid 0.05% and acetonitrile + formic acid 0.05% as phases A and B, respectively. The flow rate was set at 0.4 ml/min and the column temperature at 30 °C. An injection of 2.5 μl was made. The gradient started at 2% B, linearly increased to 40% B in 3 min, then to 100% B in 2 min, and the column was finally washed at 100% B for 2 min and reconditioned at 2% B for 4 min. The mass spectrometer was operated in MRM mode using specific transitions for each benzoxazinoid compound. The instrument was run in negative ionization from 0.0–4.28 min for the detection of DIMBOA-Glc HMBOA-Glc and MBOA-Glc, and in positive ionization from 4.29–5.0 min for the detection of HDMBOA-Glc and MBOA. The gas temperature in the MS source (TEM) was set to 550 °C and the nebulizing (GS1), drying (GS2) and curtain (CUR) gas flows set to 55, 50 and 15 psi, respectively. Quantification was done by external calibration using standards purified from plants at 1, 5, 20, 100, 500 and 2000 ng/ml. The lowest limits of quantification on pure standards were 0.5–1.0 ng/ml for benzoxazinoid glucosides and 2.0 ng/ml for benzoxazinoid aglucones.

### EPN infectivity tests

Two different experiments were conducted with the newly isolated Mexican EPN. In the first experiment, the capacity of infection of the 40 EPN isolates was evaluated to test whether WCR larvae are more resistant against EPN attack than BCB larvae. Here, both WCR and BCB larvae were fed on the hybrid DFI 45321, which contains benzoxazinoids. The second experiment aimed to test whether the differences between the infection of WCR and BCB larvae could be explained by the sequestration of benzoxazinoids by WCR larvae. For this, we assessed the infection capacity of the 24 collected *H. bacteriophora* isolates on larvae of WCR that were fed either on the benzoxazinoid-containing wild type B73 line or on its near-isogenic *bx1* mutant line. Both experiments were carried out according to Zhang *et al*.^30^. Briefly, four WCR or four BCB larvae were placed in a Ø55 mm Petri-dish (VWR International, LLC, Switzerland), that was half-filled with moist autoclaved sand (Ø 1–4 mm) (Migros, Switzerland). Approximately five hundred newly emerged infective juvenile nematodes suspended in 500 µl of tap water were applied to each Petri-dish (concentration of 21 IJs/cm^2^). Controls were treated with 500 µl of tap water. All Petri-dishes were sealed with parafilm to prevent larvae from escaping and to maintain a high level of humidity. The dishes were kept in the dark at 22 °C and EPN infection was verified 7 days after inoculation by recording colour changes (Supplementary Fig. S9) or by direct dissection. A total of 10–20 dishes for each EPN isolate were prepared for each experiment.

### Statistical analysis

All data were analysed in R (R Development Core Team 2015). Differences in benzoxazinoids contents in plants and larvae were assessed with One-way ANOVA under Gaussian error distribution. Larval infections were compared within each EPN for the different type of larvae using Generalized Linear Models (GLM) under binomial distribution. All analyses were followed by residual analysis to verify the suitability of the error distribution and model fitting when necessary to quasibinomial models.

## Supplementary information

Supplementary Information.

## Data Availability

The data supporting the findings of this study are available in the supplementary materials.
