# Supplementary material for: Entomopathogenic nematodes from Mexico that can overcome the resistance mechanisms of the western corn rootworm
Source: Sci Rep. 2020 May 19;10:8257. doi: 10.1038/s41598-020-64945-x (PMC7237494; doi:10.1038/s41598-020-64945-x)
Supplement: Supplementary file 1 — Supplementary Information. [file 41598_2020_64945_MOESM1_ESM.pdf]

Supplementary information

Entomopathogenic nematodes from Mexico that can overcome the resistance mechanisms of the western corn rootworm

Pamela Bruno, Ricardo AR Machado, Gaétan Glauser, Angela Köhler, Raquel Campos-Herrera, Julio Bernal, Stefan Toepfer, Matthias Erb, Christelle AM Robert, Carla CM Arce and Ted CJ Turlings

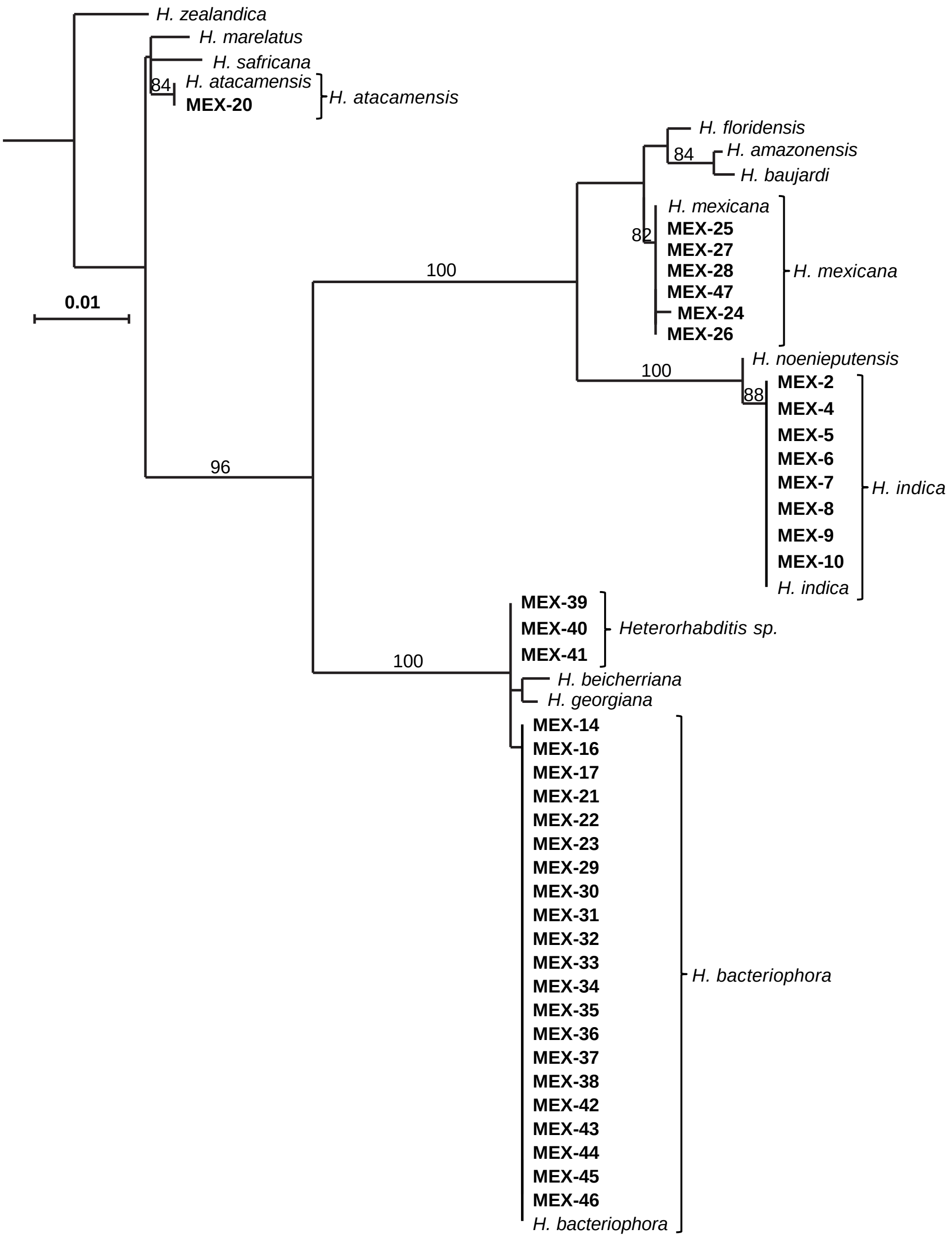

Figure S1. Maximum-likelihood phylogenetic tree of 14 species of *Heterorhabditis* nematodes based on D2/D3 ribosomal RNA gene sequences. The species *H. downesi* and *H. taysearae* could not be included since there are no available sequences in the GenBank. The evolutionary distances were computed using the Kimura 2-parameter model. The tree with the highest log likelihood (-1812.68) is shown. Numbers at nodes represent bootstrap values higher than 70% based on 100 replications. The rate variation among sites was modeled with a gamma distribution (5 categories (+G, parameter = 0.0500)). Bar represents 0.01 nucleotide substitutions per sequence position. Accession numbers of the gene sequences used for the reconstructions and those obtained in this study are available in Table S1 and S2, respectively.

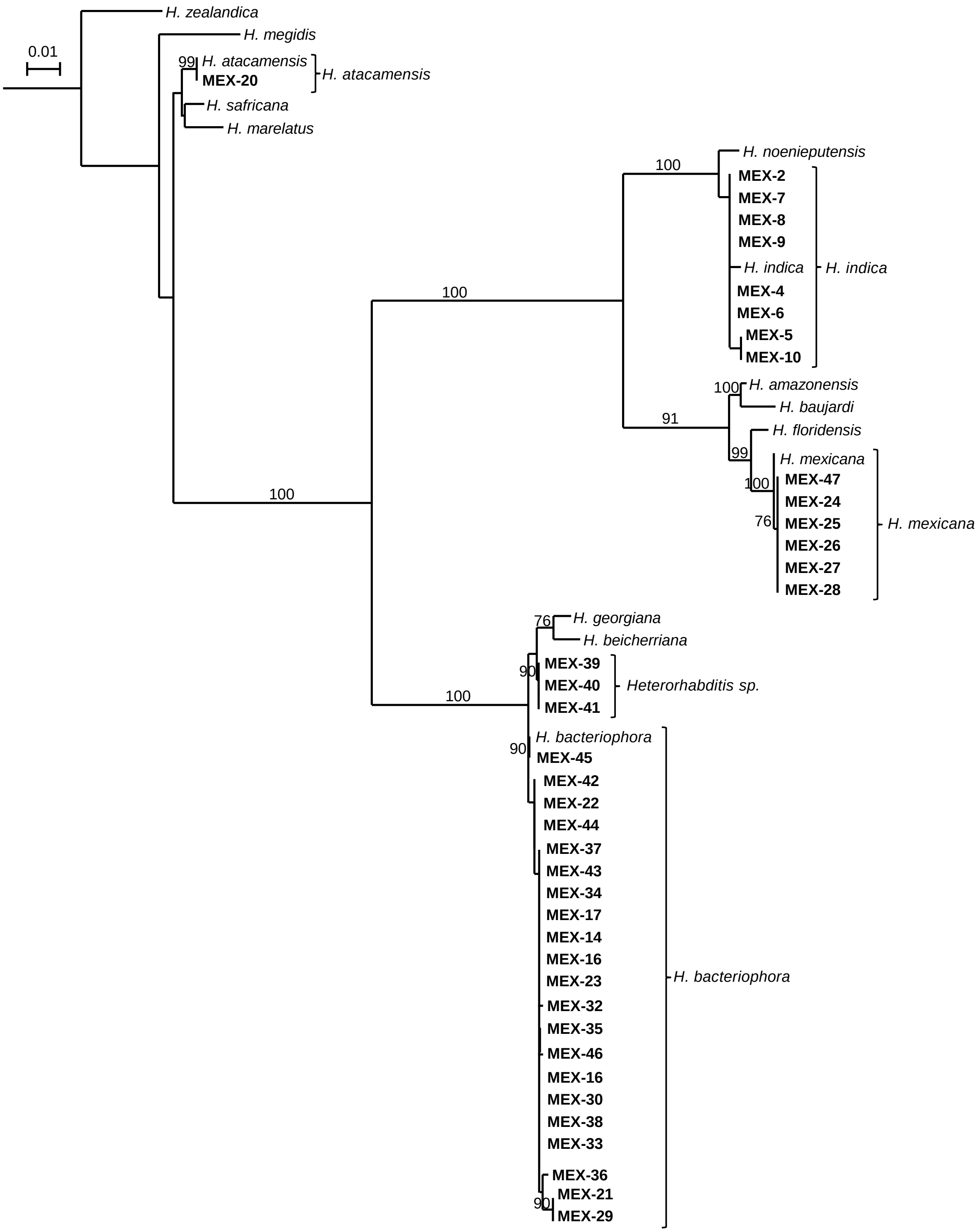

Figure S2. Maximum-likelihood phylogenetic tree of 14 species of *Heterorhabditis* nematodes based on concatenated 18S and D2/D3 ribosomal RNA gene sequences. The species *H. downesi* and *H. taysearae* could not be included since there are no available D2/D3 sequences in the GenBank. The evolutionary distances were computed using the Kimura 2-parameter model. The tree with the highest log likelihood (-6209.06) is shown. Numbers at nodes represent bootstrap values higher than 70% based on 100 replications. The rate variation among sites was modeled with a gamma distribution (5 categories (+G, parameter = 0.2726)). Bar represents 0.01 nucleotide substitutions per sequence position. Accession numbers of the gene sequences used for the reconstructions and those obtained in this study are available in Table S1 and S2, respectively.

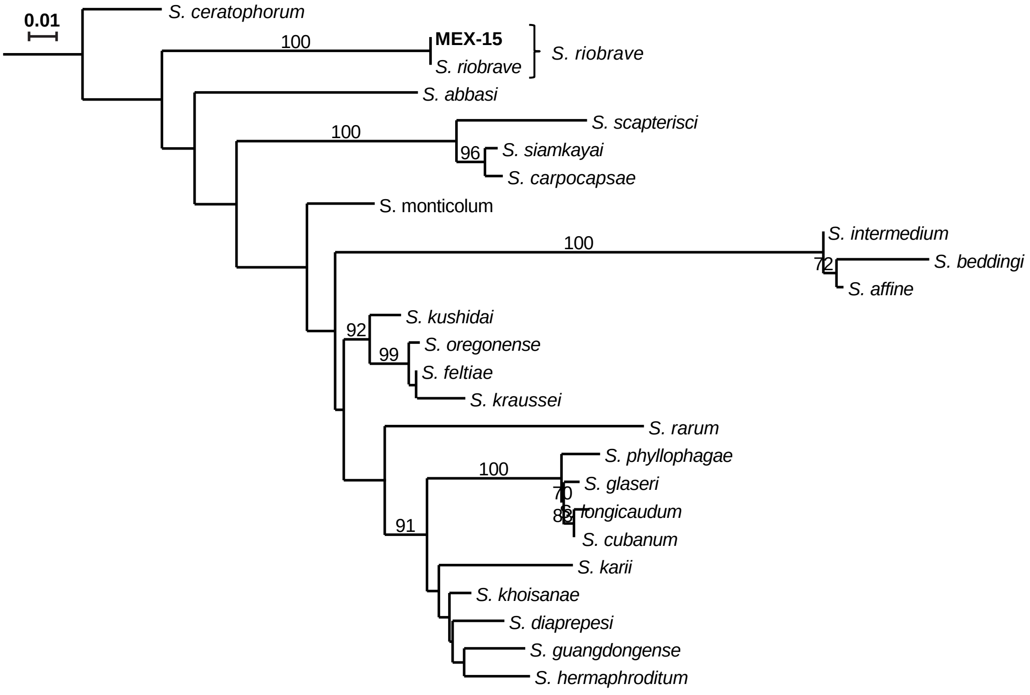

Figure S3. Maximum-likelihood phylogenetic tree of 24 species of *Steinernema* based on D2/D3 ribosomal RNA gene sequences. The evolutionary distances were computed using the Kimura 2-parameter model. The tree with the highest log likelihood (-(-4491.90) is shown. Numbers at nodes represent bootstrap values higher than 70% based on 100 replications. The rate variation among sites was modeled with a gamma distribution (5 categories (+G, parameter = 0.2931)). Bar represents 0.01 nucleotide substitutions per sequence position. Accession numbers of the gene sequences used for the reconstruction and those obtained in this study are available in Table S1 and S2, respectively.

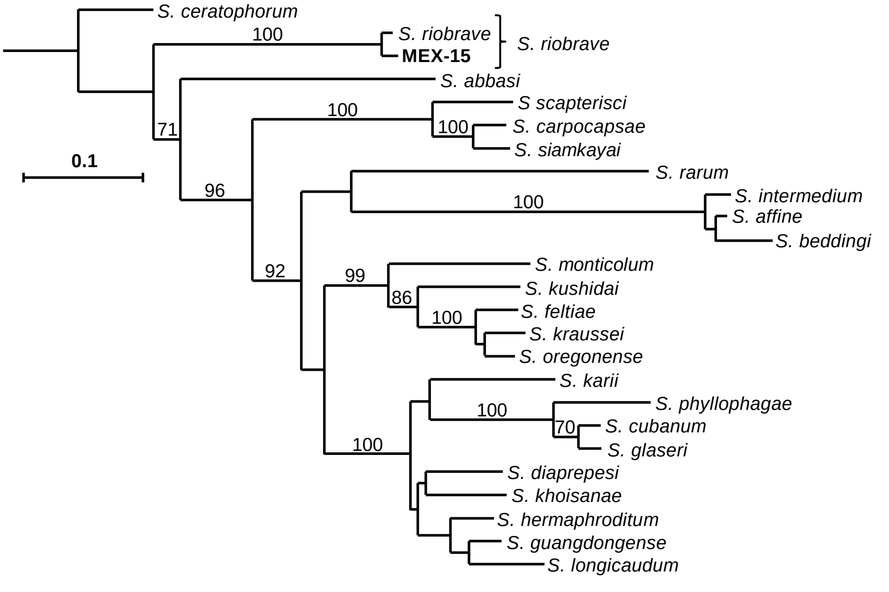

Figure S4. Maximum-likelihood phylogenetic tree of 24 species of *Steinernema* nematodes based on concatenated 18S and D2/D3 ribosomal RNA gene sequences. The evolutionary distances were computed using the Kimura 2-parameter model. The tree with the highest log likelihood (-16814.12) is shown. Numbers at nodes represent bootstrap values higher than 70% based on 100 replications. The rate variation among sites was modeled with a gamma distribution (5 categories (+G, parameter = 0.3552)). Bar represents 0.01 nucleotide substitutions per sequence position. Accession numbers of the gene sequences used for the reconstructions and those obtained in this study are available in Table S1 and S2, respectively.

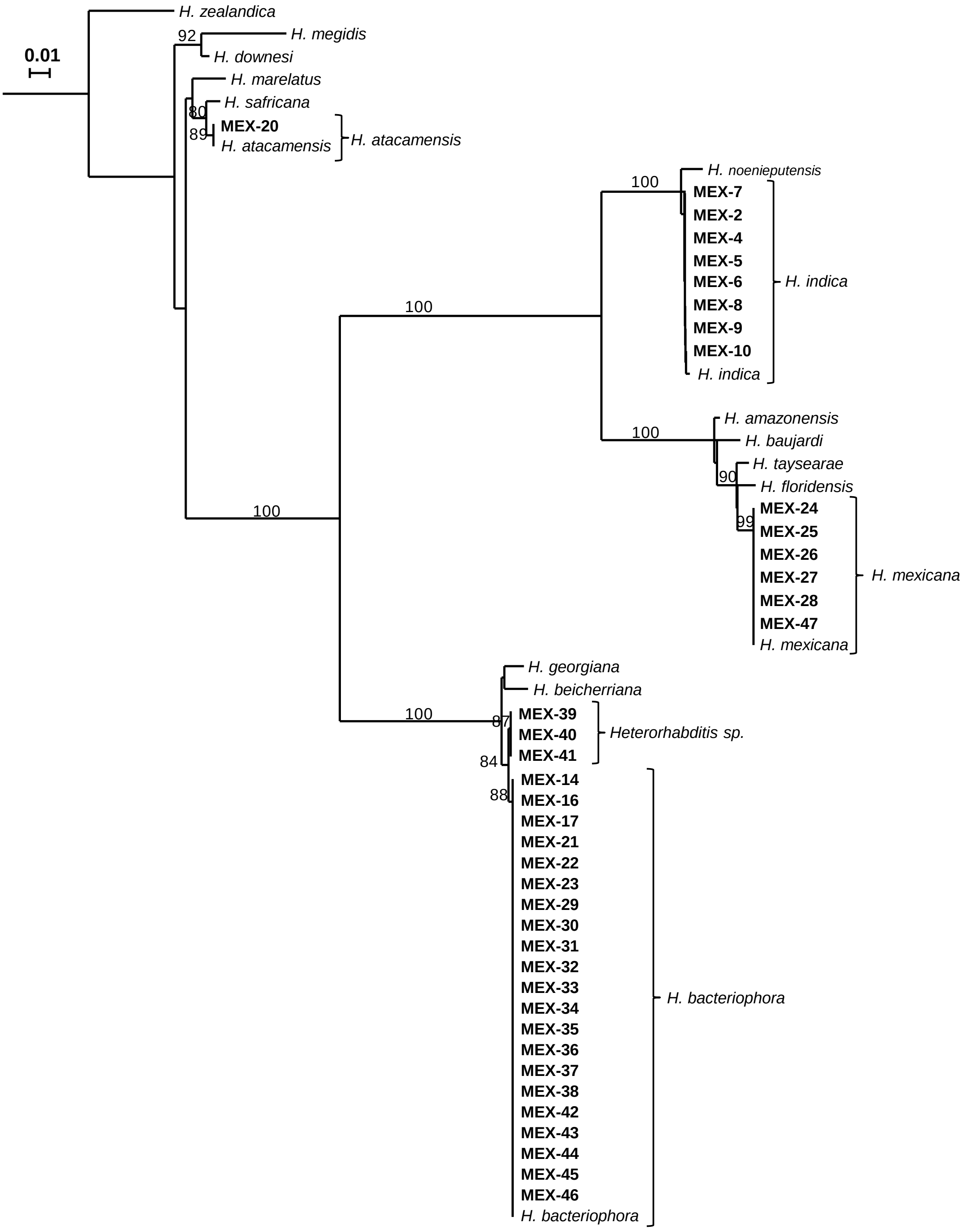

Figure S5. Neighbour-Joining phylogenetic tree of 16 species of *Heterorhabditis* nematodes based on 18S ribosomal RNA gene sequences. The evolutionary distances were computed using the Kimura 2-parameter model. Numbers at nodes represent bootstrap values higher than 70% based on 1000 replications. The rate variation among sites was modeled with a gamma distribution (shape parameter = 1). Bar represents 0.01 nucleotide substitutions per sequence position. Accession numbers of the gene sequences used for the reconstructions and those obtained in this study are available in Table S1 and S2, respectively.

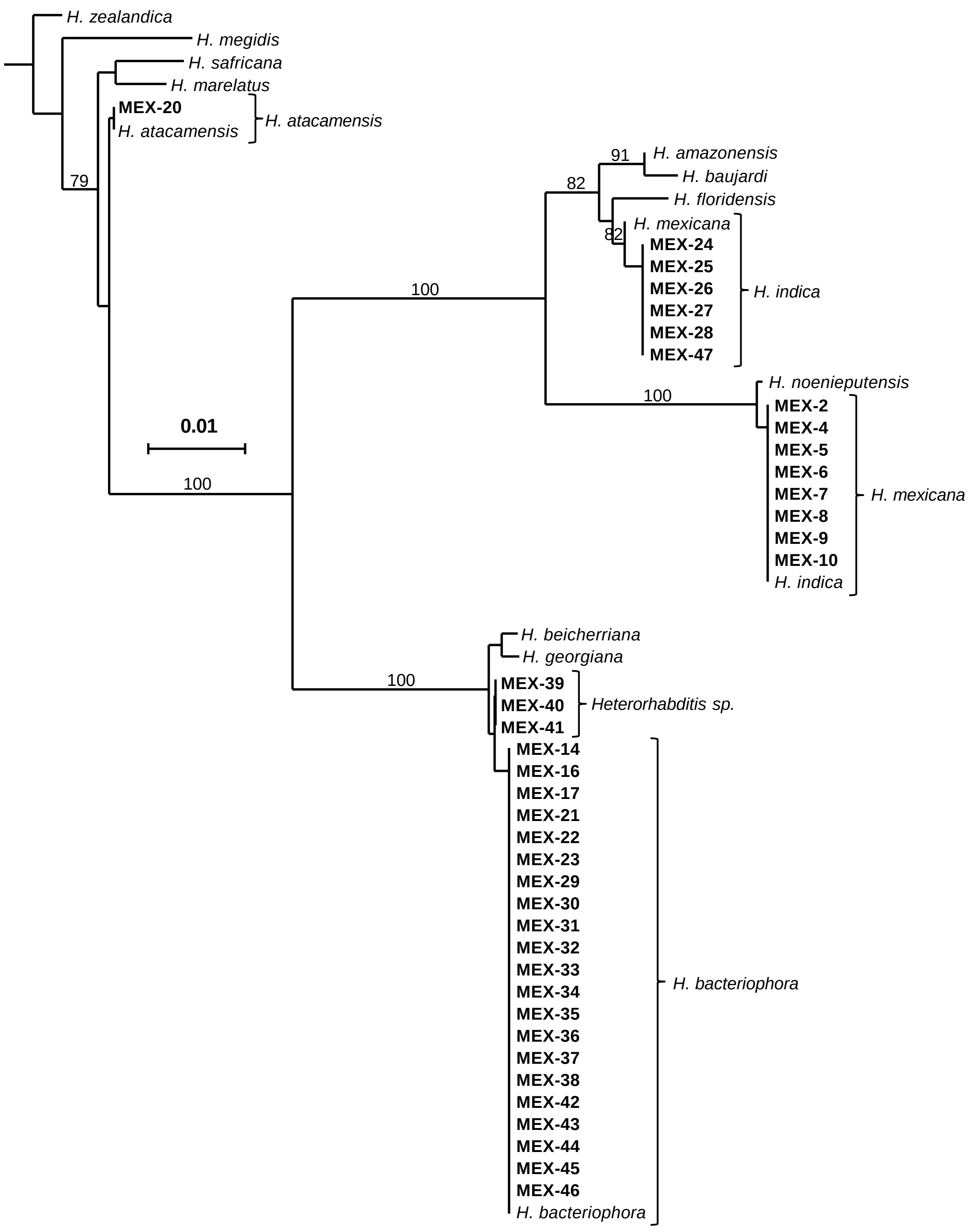

Figure S6. Neighbour-Joining phylogenetic tree of 14 species of *Heterorhabditis* nematodes based on D2/D3 ribosomal RNA gene sequences. The species *H. downesi* and *H. taysearae* could not be included since there are no available sequences in the GenBank. The evolutionary distances were computed using the Kimura 2-parameter model. Numbers at nodes represent bootstrap values higher than 70% based on 1000 replications. The rate variation among sites was modeled with a gamma distribution (shape parameter = 1). Bar represents 0.01 nucleotide substitutions per sequence position. Accession numbers of the gene sequences used for the reconstruction and those obtained in this study are available in Table S1 and S2, respectively.

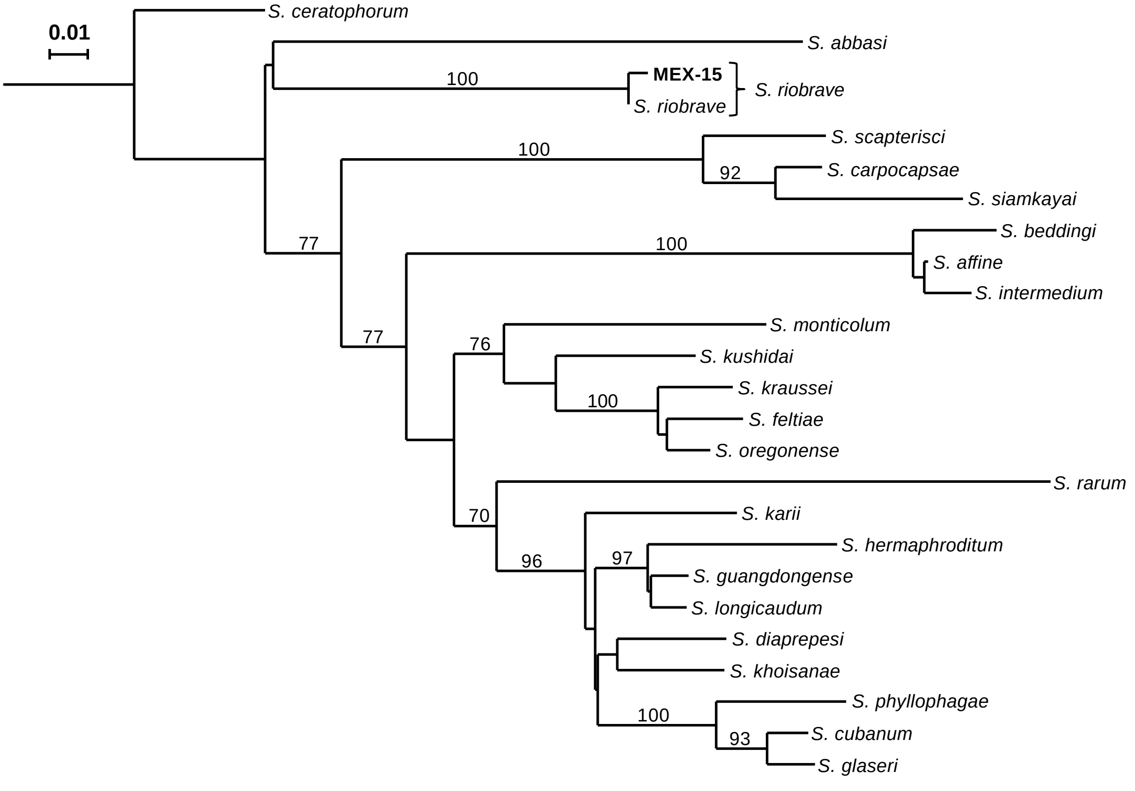

Figure S7. Neighbour-Joining phylogenetic tree of 24 species of *Steinernema* nematodes based on 18S ribosomal RNA gene sequences. The evolutionary distances were computed using the Kimura 2-parameter model. Numbers at nodes represent bootstrap values higher than 70% based on 1000 replications. The rate variation among sites was modeled with a gamma distribution (shape parameter = 1). Bar represents 0.01 nucleotide substitutions per sequence position. Accession numbers of the gene sequences used for the reconstruction and those obtained in this study are available in Table S1 and S2, respectively.

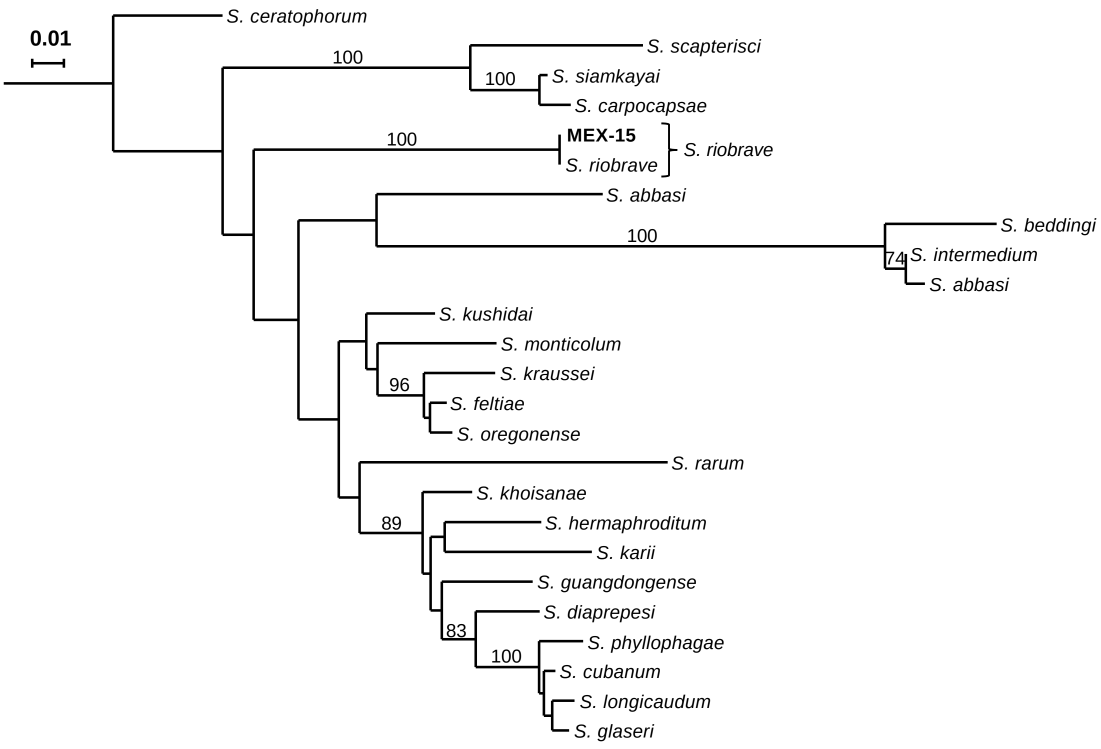

Figure S8. Neighbour-Joining phylogenetic trees of 24 species of *Steinernema* nematodes based on D2/D3 ribosomal RNA gene sequences. The evolutionary distances were computed using the Kimura 2-parameter model. Numbers at nodes represent bootstrap values higher than 70% based on 1000 replications. The rate variation among sites was modeled with a gamma distribution (shape parameter = 1). Bar represents 0.01 nucleotide substitutions per sequence position. Accession numbers of the gene sequences used for the reconstruction and those obtained in this study are available in Table S1 and S2, respectively.

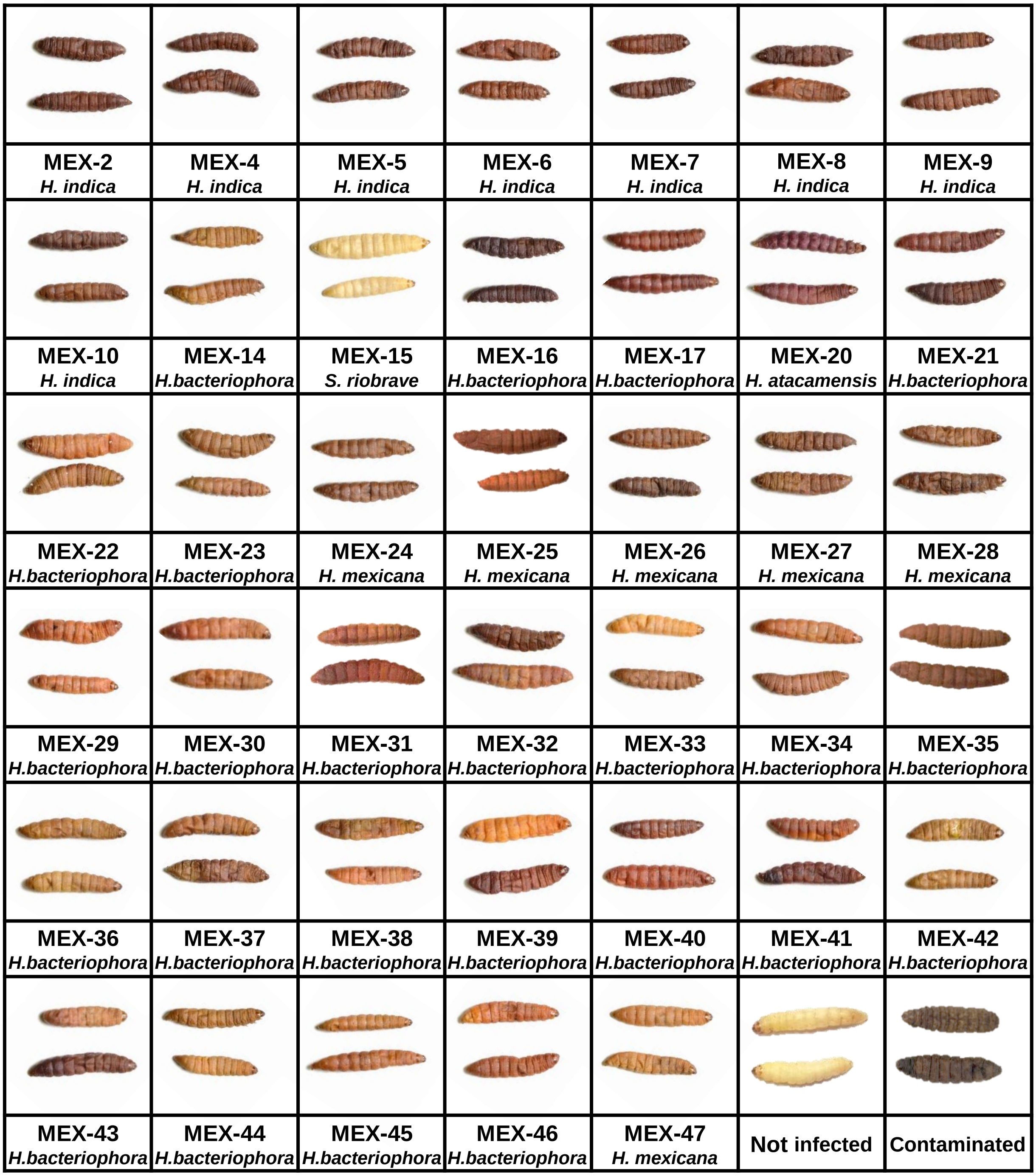

Figure S9. Coloration pattern of *Galleria mellonella* larvae infected with Mexican EPN. These colors were used as a reference for the evaluation of the infectivity tests.

Table S1. Accession numbers of the sequences used for the phylogenetic reconstructions in this study.

Reference species	Accession number	
	18s	D2/D3
Heterorhabditis amazonensis	KU870321.1	EU099036.1
Heterorhabditis atacamensis	HM230723.1	HM230724.1
Heterorhabditis bacteriophora	EU716335.2	EU313541.1
Heterorhabditis baujardi	EU363039.1	MF621013.1
Heterorhabditis beicherriana	HQ896630.2	HQ896631.2
Heterorhabditis downesi	EF043442.1	N/A
Heterorhabditis floridensis	DQ372922.1	EU099034.1
Heterorhabditis georgiana	HQ225885.1	EU099033.1
Heterorhabditis indica	MK990223.1	EU100415.1
Heterorhabditis marelatus	AY321479.1	EU100412.1
Heterorhabditis megidis	AY293284.1	EU100413.1
Heterorhabditis mexicana	EF043444.1	EU100414.1
Heterorhabditis noenieputensis	FJ235075.1	JX624110.1
Heterorhabditis safricana	FJ473361.1	EU100416.1
Heterorhabditis taysearae	EF043443.1	N/A
Heterorhabditis zealandica	GU174010.1	EU099035.1
Steinernema abbasi	KF573496.1	MG198916.1
Steinernema affine	KY818705.1	KC287226.2
Steinernema beddingi	AY603397.1	AY603396.1
Steinernema carpocapsae	KC571265.1	KJ950293.1
Steinernema ceratophorum	AY230165.1	AF331888.1
Steinernema cubanum	AY230166	AF331889.1
Steinernema diaprepesi	AF122021.1	DQ849320.1
Steinernema feltiae	AY230169.1	JF728855.1
Steinernema glaseri	AY230171.1	AF331908.1
Steinernema guangdongense	AY170341.1	AY169558.1
Steinernema hermaphroditum	JQ687355.1	JQ687356.1
Steinernema intermedium	AY230172.1	JN808125.1
Steinernema karii	GQ497742.1	AF331902.1
Steinernema khoisanae	DQ314287.1	DQ314289.1
Steinernema kraussei	AY230175.1	JN683826.1
Steinernema kushidai	AB243440.1	AF331897.1
Steinernema longicaudum	AY230177.1	AF331894.1
Steinernema monticolum	AF122017.1	HM778114.1
Steinernema oregonense	AY230180.1	AF331891.1
Steinernema rarum	DQ221117.1	KT378447.1
Steinernema riobrave	GU174001.1	AF331893.1
Steinernema scapterisci	AF122020.1	GU395646.1
Steinernema siamkayai	KX886343.1	KY311815.1
Steinernema phyllophagae	FJ410327.1	FJ666054.1

Table S2. Accession numbers of the sequences obtained in this study and deposited to the GenBank.

Isolate	Proposed classification	Accession number	
		18s	D2/D3
MEX-2	*Heterorhabditis indica*	MK421471	MK421431
MEX-4	*Heterorhabditis indica*	MK421473	MK421433
MEX-5	*Heterorhabditis indica*	MK421474	MK421434
MEX-6	*Heterorhabditis indica*	MK421475	MK421435
MEX-7	*Heterorhabditis indica*	MK421476	MK421436
MEX-8	*Heterorhabditis indica*	MK421477	MK421437
MEX-9	*Heterorhabditis indica*	MK421478	MK421438
MEX-10	*Heterorhabditis indica*	MK421479	MK421439
MEX-14	*Heterorhabditis bacteriophora*	MK421482	MK421440
MEX-15	*Steinernema riobrave*	MK421550	MK421555
MEX-16	*Heterorhabditis bacteriophora*	MK421483	MK421441
MEX-17	*Heterorhabditis bacteriophora*	MK421484	MK421442
MEX-20	*Heterorhabditis atacamensis*	MK421485	MK421443
MEX-21	*Heterorhabditis bacteriophora*	MK421486	MK421444
MEX-22	*Heterorhabditis bacteriophora*	MK421487	MK421445
MEX-23	*Heterorhabditis bacteriophora*	MK421488	MK421446
MEX-24	*Heterorhabditis mexicana*	MK421489	MK421447
MEX-25	*Heterorhabditis mexicana*	MK421490	MK421448
MEX-26	*Heterorhabditis mexicana*	MK421491	MK421449
MEX-27	*Heterorhabditis mexicana*	MK421492	MK421450
MEX-28	*Heterorhabditis mexicana*	MK421493	MK421451
MEX-29	*Heterorhabditis bacteriophora*	MK421494	MK421452
MEX-30	*Heterorhabditis bacteriophora*	MK421495	MK421453
MEX-31	*Heterorhabditis bacteriophora*	MK421496	MK421454
MEX-32	*Heterorhabditis bacteriophora*	MK421497	MK421455
MEX-33	*Heterorhabditis bacteriophora*	MK421498	MK421456
MEX-34	*Heterorhabditis bacteriophora*	MK421499	MK421457
MEX-35	*Heterorhabditis bacteriophora*	MK421500	MK421458
MEX-36	*Heterorhabditis bacteriophora*	MK421501	MK421459
MEX-37	*Heterorhabditis bacteriophora*	MK421502	MK421460
MEX-38	*Heterorhabditis bacteriophora*	MK421503	MK421461
MEX-39	*Heterorhabditis bacteriophora*	MK421504	MK421462
MEX-40	*Heterorhabditis bacteriophora*	MK421505	MK421463
MEX-41	*Heterorhabditis bacteriophora*	MK421506	MK421464
MEX-42	*Heterorhabditis bacteriophora*	MK421507	MK421465
MEX-43	*Heterorhabditis bacteriophora*	MK421508	MK421466
MEX-44	*Heterorhabditis bacteriophora*	MK421509	MK421467
MEX-45	*Heterorhabditis bacteriophora*	MK421510	MK421468
MEX-46	*Heterorhabditis bacteriophora*	MK421511	MK421469
MEX-47	*Heterorhabditis mexicana*	MK421512	MK421470

Table S3 ANOVA results for the comparisons of benzoxazinoids present in WCR and BCB larvae fed on hybrid maize DFI 45321 (*n*=5). Stars indicate significant differences between treatments: *p<0.05, **p<0.01, ***p<0.001.

Benzoxazinoids	WCR larvae fed on hybrid maize DFI 45321		BCB larvae fed on hybrid maize DFI 45321		F	p-value	Signif. codes
	Mean (µg/g FW)	±SE	Mean (µg/g FW)	±SE			
MBOA	2.75	0.74	0.12	0.03	12.591	7.53E-03	**
MBOA-Glc	11.04	0.77	1.56	0.59	95.521	1.01E-05	***
HMBOA-Glc	27.33	3.99	1.87	0.57	40.000	2.27E-04	***
HDMBOA-Glc	476.53	58.07	1.03	0.63	67.044	3.69E-05	***
DIMBOA-Glc	7.09	2.32	0.34	0.09	8.430	1.98E-02	*
**TOTAL**	**524.73**	**65.89**	**4.92**	**1.90**	**65.585**	4.00E-05	***

Table S4 ANOVA results for the comparisons of benzoxazinoids present in maize seedlings (*n*=5). Stars indicate significant differences between treatments: *p<0.05, **p<0.01, ***p<0.001.

**Benzoxazinoids**	**WT B73**		**Mutant *bx1***		**F**	**p-value**	**Signif. codes**
	**Mean (µg/g FW)**	**±SE**	**Mean (µg/g FW)**	**±SE**			
MBOA	15.11	2.09	6.46	0.59	15.871	4.04E-03	**
MBOA-Glc	1.14	0.20	0.40	0.01	13.165	6.70E-03	**
HMBOA-Glc	29.13	7.74	3.69	0.54	10.747	1.12E-02	*
HDMBOA-Glc	159.84	43.78	9.04	3.08	11.806	8.88E-03	**
DIMBOA-Glc	380.74	73.41	16.72	2.17	24.570	1.11E-03	**
**TOTAL**	**585.96**	**127.22**	**36.31**	**6.40**	**19.922**	**2.10E-03**	**

Table S5 ANOVA results for the comparisons of benzoxazinoids present in benzoxazinoid-containing (*n*=4) and benzoxazinoid-free (*n*=5) WCR larvae. Stars indicate significant differences between treatments: *p<0.05, **p<0.01, ***p<0.001.

Benzoxazinoids	Benzoxazinoid-containing WCR larvae		Benzoxazinoid-free WCR larvae		F	p-value	Signif. codes
	Mean (µg/g FW)	±SE	Mean (µg/g FW)	±SE			
MBOA	10.88	3.27	9.69E-04	3.58E-04	14.313	6.86E-03	**
MBOA-Glc	50.96	9.54	1.14E-02	1.19E-03	36.997	5.00E-04	***
HMBOA-Glc	70.70	16.85	1.65E-02	4.31E-03	22.804	2.02E-03	**
HDMBOA-Glc	459.11	76.98	2.75E-01	4.40E-02	46.054	2.56E-04	***
DIMBOA-Glc	20.00	8.14	2.44E-03	1.61E-03	7.820	2.67E-02	*
**TOTAL**	**611.64**	**114.78**	**0.306**	**0.051**	**45.328**	**2.69E-04**	*****

Table S6 ANOVA results for the comparisons of Mexican EPN infection on WCR and BCB larvae seven days after inoculation. Stars indicate significant differences between treatments for each EPN isolate: *p<0.05, **p<0.01, ***p<0.001.

EPN isolate	Species	WCR larvae			BCB larvae			Test	p-value	Signif. code
		n	Mean (μg/g FW)	±SE	*n*	Mean (μg/g FW)	±SE			
MEX-2	*H. indica*	23	91.30	3.37	20	98.75	1.25	Chisq= 31.320	0.01896	*
MEX-4	*H. indica*	22	94.32	2.82	20	98.75	1.25	Chisq= 25.244	0.0963	n.s.
MEX-5	*H. indica*	23	82.25	6.40	10	100.00	0.00	F= 7.316	0.011	*
MEX-6	*H. indica*	23	88.41	4.74	20	100.00	0.00	Chisq= 42.333	0.001389	**
MEX-7	*H. indica*	23	88.04	4.25	20	98.75	1.25	Chisq= 36.581	0.004506	**
MEX-8	*H. indica*	25	97.00	2.20	26	99.04	0.96	Chisq= 23.308	0.2572	n.s.
MEX-9	*H. indica*	23	90.94	3.81	20	100.00	0.00	Chisq= 27.865	0.01235	**
MEX-10	*H. indica*	25	91.00	2.84	26	99.04	0.96	Chisq= 29.283	0.00358	**
MEX-14	*H. bacteriophora*	35	46.43	6.20	20	92.50	3.19	F= 29.879	1.261E-06	***
MEX-15	*S. riobrave*	23	8.70	4.04	20	100.00	0.00	Chisq= 35.799	<2.2E-16	***
MEX-16	*H. bacteriophora*	37	85.59	4.30	26	98.08	1.33	F= 7.4	0.008486	**
MEX-17	*H. bacteriophora*	20	90.00	3.80	21	92.86	3.51	F= 0.282	0.5985	n.s.
MEX-20	*H. atacamensis*	38	94.52	1.99	26	89.42	4.20	F= 1.471	0.2297	n.s.
MEX-21	*H. bacteriophora*	25	80.33	4.22	16	100.00	0.00	Chisq= 31.225	4.68E-06	***
MEX-22	*H. bacteriophora*	37	38.96	6.00	26	93.27	2.96	F= 46.469	4.779E-09	***
MEX-23	*H. bacteriophora*	37	43.02	6.17	26	92.31	3.03	F= 36.599	9.642E-08	***
MEX-24	*H. mexicana*	37	53.83	5.84	26	97.12	2.12	F= 38.689	4.972E-08	***
MEX-25	*H. mexicana*	27	38.89	6.58	20	99.00	1.00	F= 60.065	7.982E-10	***
MEX-26	*H. mexicana*	37	42.57	4.83	26	92.63	3.90	F= 44.044	9.724E-09	***
MEX-27	*H. mexicana*	37	59.01	5.22	26	87.50	5.59	F= 11.606	0.001169	**
MEX-28	*H. mexicana*	37	44.14	4.66	26	100.00	0.00	Chisq= 57.004	<2.2E-16	***
MEX-29	*H. bacteriophora*	37	35.59	5.98	26	95.83	2.41	F= 54.740	4.775E-10	***
MEX-30	*H. bacteriophora*	35	46.19	6.07	20	96.25	2.05	F= 40.817	4.331E-08	***
MEX-31	*H. bacteriophora*	27	79.01	5.54	20	98.75	1.25	F= 12.468	0.0009686	***
MEX-32	*H. bacteriophora*	20	88.75	3.84	22	88.26	4.92	F= 0.002	0.9693	n.s.
MEX-33	*H. bacteriophora*	37	46.62	6.58	26	99.04	0.96	F= 60.083	1.179E-10	***
MEX-34	*H. bacteriophora*	37	44.23	6.50	26	91.99	3.41	F= 31.128	5.877E-07	***
MEX-35	*H. bacteriophora*	35	36.67	6.58	20	68.75	7.67	F= 9.543	0.003193	**
MEX-36	*H. bacteriophora*	35	49.52	7.04	20	86.58	5.62	F= 12.850	0.0007348	***
MEX-37	*H. bacteriophora*	23	36.23	7.33	10	69.17	12.24	F= 6.063	0.01956	*
MEX-38	*H. bacteriophora*	20	70.00	5.90	22	87.50	3.58	F= 7.225	0.01043	*
MEX-39	*H. bacteriophora*	37	77.70	4.96	26	87.50	4.65	F= 1.558	0.2167	n.s.
MEX-40	*H. bacteriophora*	34	72.30	5.70	20	95.00	2.29	F= 11.209	0.00152	**
MEX-41	*H. bacteriophora*	36	85.88	3.94	26	95.19	1.97	Chisq= 65.364	0.5557	n.s.
MEX-42	*H. bacteriophora*	35	51.19	6.04	20	97.50	1.72	F= 40.419	4.86E-08	***
MEX-43	*H. bacteriophora*	35	65.71	6.28	20	80.83	8.11	F= 2.2622	0.1385	n.s.
MEX-44	*H. bacteriophora*	35	46.67	7.09	20	87.08	4.34	F= 17.010	0.0001317	***
MEX-45	*H. bacteriophora*	35	50.71	6.36	20	75.83	7.81	F= 6.307	0.01511	*
MEX-46	*H. bacteriophora*	37	39.77	6.13	26	86.86	4.36	F= 27.438	2.126E-06	***
MEX-47	*H. mexicana*	34	65.93	4.98	16	95.31	4.69	F= 12.050	0.001106	**

Table S7 ANOVA results of *H. bacteriophora* isolates infection on benzoxazinoid-containing and benzoxazinoid-free WCR larvae seven days after inoculation. Stars indicate significant differences between treatments for each EPN isolate: *p<0.05, **p<0.01, ***p<0.001.

EPN isolate	Benzoxazinoid-containing WCR larvae			Benzoxazinoid-free WCR larvae			Test	p-value	Signif. code
	n	Mean (µg/g FW)	±SE	*n*	Mean (µg/g FW)	±SE			
MEX-14	10	87.50	6.72	15	93.33	4.54	Chisq= 24.166	0.212	n.s.
MEX-16	10	97.50	2.50	15	100.00	0.00	Chisq= 4.696	0.163	n.s.
MEX-17	10	97.50	2.50	15	100.00	0.00	Chisq= 4.854	0.181	n.s.
MEX-21	10	97.50	2.50	15	100.00	0.00	Chisq= 4.854	0.174	n.s.
MEX-22	10	90.00	4.08	15	91.11	4.18	Chisq= 22.895	0.796	n.s.
MEX-23	10	77.50	5.83	15	93.33	2.95	Chisq= 20.466	0.022	*
MEX-29	10	70.00	5.85	15	85.56	4.41	Chisq= 22.005	0.108	n.s.
MEX-30	10	83.33	6.80	15	94.44	3.01	Chisq= 21.804	0.100	n.s.
MEX-31	10	100.00	0.00	15	95.00	3.62	Chisq= 13.675	0.078	n.s.
MEX-32	10	97.50	2.50	15	98.21	1.79	Chisq= 10.337	0.796	n.s.
MEX-33	10	69.17	10.40	15	84.44	4.87	F= 2.012	0.169	n.s.
MEX-34	10	90.00	4.08	15	98.33	1.67	Chisq= 13.403	0.062	n.s.
MEX-35	10	92.50	3.82	15	89.29	5.05	F= 0.266	0.611	n.s.
MEX-36	10	67.50	10.57	15	86.11	6.12	F= 3.014	0.096	n.s.
MEX-37	10	65.00	7.64	15	89.88	5.81	F= 7.715	0.011	*
MEX-38	10	91.67	4.30	15	87.78	4.29	Chisq= 22.631	0.410	n.s.
MEX-39	10	95.00	5.00	15	100.00	0.00	Chisq= 6.422	0.163	n.s.
MEX-40	10	92.50	3.82	15	100.00	0.00	Chisq= 7.815	0.025	*
MEX-41	10	100.00	0.00	15	95.00	3.62	Chisq= 10.128	0.145	n.s.
MEX-42	10	100.00	0.00	15	93.33	3.83	Chisq= 14.849	0.047	*
MEX-43	10	86.67	4.51	15	90.56	3.62	Chisq= 18.781	0.559	n.s.
MEX-44	10	87.50	6.72	15	95.00	2.67	Chisq= 22.547	0.367	n.s.
MEX-45	10	92.50	5.34	15	85.56	5.04	F= 0.521	0.478	n.s.
MEX-46	10	80.00	6.24	15	86.11	5.73	F= 1.224	0.280	n.s.
